# The inoculum dose of Zika virus can affect the viral replication dynamics, cytokine responses and survival rate in immunocompromised AG129 mice

**DOI:** 10.1186/s43556-024-00195-x

**Published:** 2024-08-03

**Authors:** Yuhuan Yan, Hao Yang, Yun Yang, Junbin Wang, Yanan Zhou, Cong Tang, Bai Li, Qing Huang, Ran An, Xiaoming Liang, Dongdong Lin, Wenhai Yu, Changfa Fan, Shuaiyao Lu

**Affiliations:** 1 Institute of Medical Biology, Chinese Academy of Medical Sciences & Peking Union Medical College, Kunming, 650118 China; 2 National Rodent Laboratory Animal Resources Center, Institute for Laboratory Animal Resources, National Institutes for Food and Drug Control (NIFDC), Beijing, 102629 China; 3 Key Laboratory of Pathogen Infection Prevention and Control (Peking Union Medical College), Ministry of Education, Beijing, China; 4 State Key Laboratory of Respiratory Health and Multimorbidity, Beijing, China; 5 Yunnan Provincial Key Laboratory of Vector-Borne Diseases Control and Research, Kunming, China

**Keywords:** Zika virus, AG129 mouse, Replication dynamics, Tissue tropism, Pathological lesion, Cytokine

## Abstract

Zika virus, a mosquito-borne arbovirus, has repeatedly caused large pandemics with symptoms worsening from mild and self-limiting diseases to Guillain–Barré syndrome in adults and fetal microcephaly in newborns. In recent years, Zika virus diseases have posed a serious threat to human health. The shortage of susceptible small animal models makes it difficult to study pathogenic mechanisms and evaluate potential therapies for Zika virus infection. Therefore, we chose immunocompromised mice (AG129 mice) deficient in IFN-α/β and IFN-γ receptors, which can abolish the innate immune system that prevents Zika virus infection early. AG129 mice were infected with the Zika virus, and this mouse model exhibited replication dynamics, tissue tropism, pathological lesion and immune activation of the Zika virus. Our results suggest that the inoculum dose of Zika virus can affect the viral replication dynamics, cytokine responses and survival rate in AG129 mice. By testing the potential antiviral drug favipiravir, several critical indicators, including replication dynamics and survival rates, were identified in AG129 mice after Zika virus infection. It is suggested that the model is reliable for drug evaluation. In brief, this model provides a potential platform for studies of the infectivity, virulence, and pathogenesis of the Zika virus. Moreover, the development of an accessible mouse model of Zika virus infection will expedite the research and deployment of therapeutics and vaccines.

**Supplementary Information:**

The online version contains supplementary material available at 10.1186/s43556-024-00195-x.

## Introduction

Zika virus (ZIKV) is a mosquito-borne arbovirus that was first isolated from a sentinel rhesus macaque in Uganda in 1947 during studies of enzootic yellow fever [1]. The genome organization of ZIKV is significantly similar to that of other flaviviruses such as dengue virus, West Nile virus, and yellow fever virus. ZIKV has a single-stranded positive-sense and 11 kilobase RNA genome encoding three structural proteins [capsid (C), precursor membrane (prM), and envelope (E)] and seven nonstructural proteins (NS1, NS2A, NS2B, NS3, NS4A, NS4B, and NS5) [2].

ZIKV was once considered an obscure member of the mosquito-borne flavivirus family for six decades, and human infections with ZIKV were thought to be sporadic, with mild and self-limiting symptoms [3, 4]. However, the first large outbreak emerged on Yap Island, Micronesia, in 2007, with 49 confirmed and 59 probable cases of Zika virus disease [5]. In 2013, there was a larger ZIKV outbreak in French Polynesia [6]. A total of 29,000 people were estimated to have been infected, at least 396 confirmed ZIKV cases were reported [7], and more than 50 cases [8]of Guillain–Barré syndrome (GBS) were linked to ZIKV infection. This is the first study providing evidence for ZIKV infection causing GBS. These data indicate the increased virulence of the ZIKV strain. The earliest confirmed cases of ZIKV infection in the Americas were identified in May 2015 [9]. Then, ZIKV quickly spread, and at least 48 countries and territories in the Americas were affected by the virus by the end of 2016 [10]. Moreover, increasing clinical evidence has shown that ZIKV infection is associated with congenital malformations, including microcephaly, intrauterine growth restriction, and fetal demise [3, 11]. This led the World Health Organization to declare a public health emergency of international concern in 2016 [12]. Although there has been no ongoing Zika virus outbreak around the world since 2017 [13], new research suggests that a single amino acid substitution in the Zika virus genome may increase infectivity, transmission, and pathogenesis, which further highlights the necessity of preparing for another ZIKV outbreak [14–16].

The development of animal models is indispensable for future epidemic responses. Research models of ZIKV infection include in vitro and in vivo models. In vitro models are essential for studying the life cycle, infection mechanism, and cytopathic effect of the Zika virus. Some 2D cell lines are available, manageable, and inexpensive. However, most cell lines have been genetically modified for immortalization and may not display an accurate molecular profile of ZIKV infection. Therefore, the results must be interpreted prudently because some modifications can easily impact the pathogenicity of the virus and yield inconsistent experimental results [17]. Models involving three-dimensional (3D) organoids have gradually replaced the 2D monolayer models. Organoids resemble in vivo conditions more than 2D monolayer cell cultures [18]. The number of cases of microcephaly has increased considerably with the increase in the number of cases of ZIKV infection in Brazil. Scientists have examined the effects of ZIKV infection on human neural stem cells growing as neurospheres and brain organoids, and direct evidence has emerged that ZIKV can infect and cause the death of neural stem cells [19]. However, the use of organoids as disease models has several limitations. Current organoid systems have reproducibility concerns as researchers cannot control how cells self-organize into organoids. Existing 3D organoid models are still unable to recapitulate the complex and dynamic inflammatory process that occurs in vivo [18]. In vivo animal models could be used to study the vertical transmission, immune activation, and tissue tropism of ZIKV. Animal models of ZIKV infection involving neuropathology and immunopathology based on nonhuman primates (NHPs) have been studie [20, 21]. Although NHPs have many advantages because of their close resemblance to humans, their use in preclinical studies is limited because of ethical issues and high breeding costs. Another more commonly available species is the domestic pig which shares genetic, physiologic, immunologic, and anatomical features with humans. Another advantage of pigs is that they carry many fetuses. The susceptibility of newborn piglets to ZIKV infection has been reported [22]. However, the large size and long feeding time of pigs cause considerable inconvenience in scientific research. The development of a high-throughput small animal model of ZIKV infection could expedite the testing and screening of drugs and vaccines before more costly evaluation in nonhuman primate and human clinical trials.

Indeed, despite much anticipation of candidate ZIKV vaccines, there have been no pivotal phase III trials demonstrating their safety and efficacy since the incidence of Zika virus cases peaked in early 2016 [23]. Researchers need to continue developing animal models of ZIKV infection. Reportedly, wild-type mice are not readily infected by ZIKV. One reason for this is that viral NS5 proteins can bind and degrade human STAT2 in the antiviral IFN signaling pathway but not mouse STAT2, which enables ZIKV to antagonize type I IFN in humans but not in mice [24–26]. Therefore, mice with genetic deficiencies in IFN-I signaling have been developed for ZIKV research. The lack of IFN-α/β and IFN-γ receptors renders AG129 mouse models incapable of responding to type I IFN and type II IFN, and these models have been used successfully to characterize ZIKV infection [27]. Here, we inoculated AG129 mice with the ZIKV strain GZ01 to characterize the infection characteristics, viral dynamics, tissue tropism, pathological features and immune activation of the Zika virus. Moreover, we selected the antiviral drugs favipiravir and testosterone to assess the reliability of the AG129 mouse model, which could contribute to a more comprehensive presentation of this model.

## Results

### ZIKV infection caused clinical symptoms and viremia in AG129 mice

**Fig. 1:**
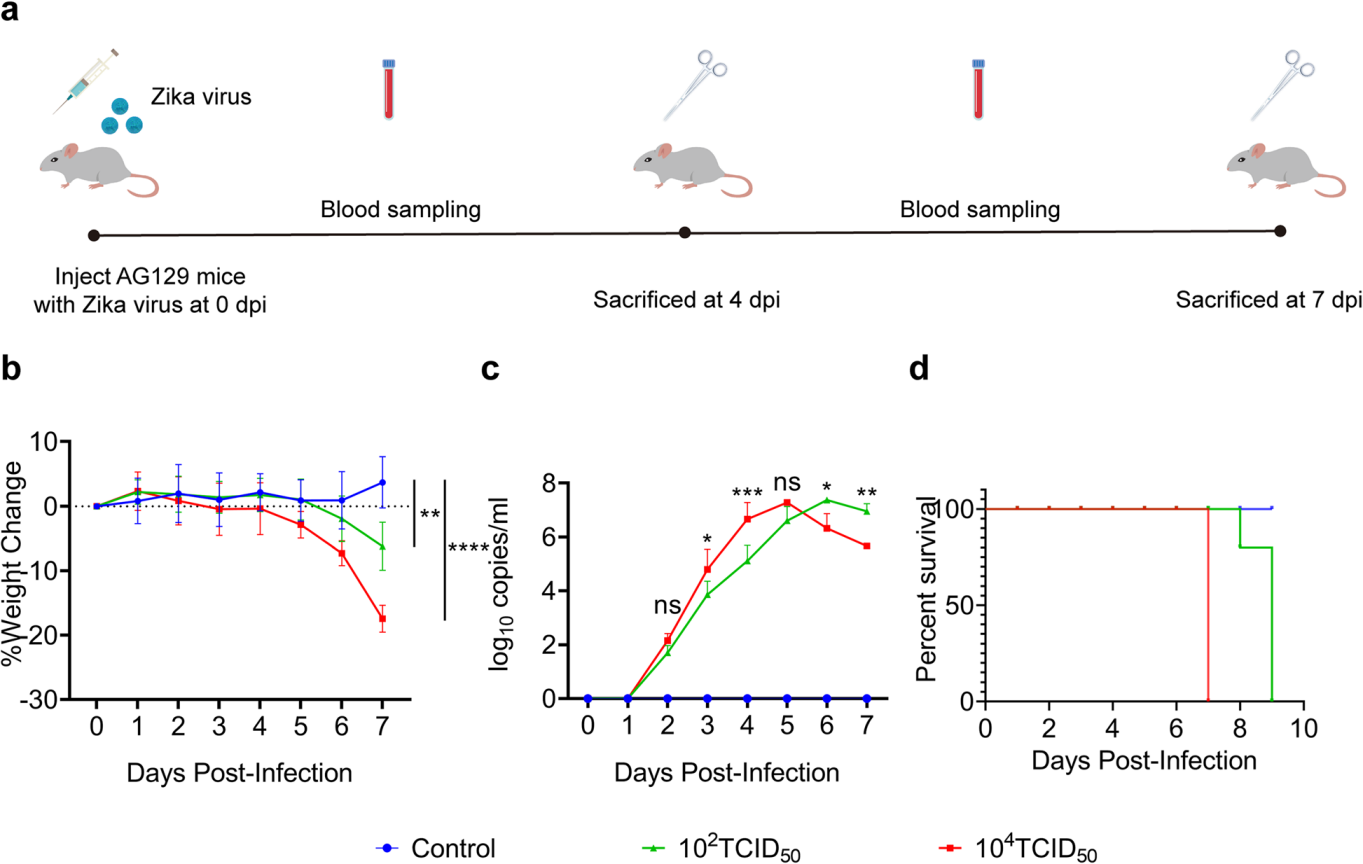
ZIKV infection caused clinical symptoms and viremia in AG129 mice. **a** Schematic illustration of AG129 mouse model infected with ZIKV GZ01 strain (related mice elements are designed by Freepik); **b** Line chart of weight change of AG129 male mice measured from 0 dpi to 7 dpi (*n* = 6); **c** Line chart of viral load in blood of AG129 male mice measured from 0 to 7 dpi (*n* = 6); **d** Survival percentage for AG129 male mice infected with ZIKV (1*10^2^ TCID_50_ and 1*10^4^ TCID_50_) (*n* = 5). Quantitative data are shown as the mean ± SD (error bars). *P < 0.05, **P < 0.01, ***P < 0.001, ****P < 0.0001 (Sidak's multiple comparisons test)

To validate that AG129 mice were susceptible to ZIKV and to determine the viral dose required to achieve robust ZIKV infection, we infected 6 ~ 8-week-old mice by subcutaneous injection of two different doses of ZIKV (GZ01). The experimental design is summarized in (Fig. 1a). In our results similar to those of a previous report [28], AG129 mice were vulnerable to ZIKV infection, began to exhibit ruffled fur, lethargy and a hunched posture at 3–4 days post infection (dpi), and deteriorated rapidly. Mice in 10^2^ TCID_50_ group became immobile and weak at 7 dpi and died over the next two days, while mice in 10^4^ TCID_50_ group died at 7dpi (Fig. 1d). After infection, we monitored the temperature and weight daily to measure mouse morbidity and detected the viral load based on blood samples collected from the tail vein. Compared to those in the control group, there were no regular changes in the body temperature fluctuations of these two groups of infected mice (Fig. S1). However, body weight tended to decrease in the infected groups (Fig. 1b). The ZIKV-challenged AG129 mice started to lose weight rapidly on the third day after infection, and mice in the 10^4^ TCID_50_ group lost weight significantly faster than those in the 10^2^ TCID_50_ group (Fig. 1b). Moreover, we detected the viral load in mouse blood as early as the second day after infection (Fig. 1c). The viral load in the blood of the 10^4^ TCID_50_ group peaked at 5 dpi, and that of the 10^2^ TCID_50_ group peaked at 6 dpi (Fig. 1c). These results showed that AG129 mice were successfully infected with ZIKV and that 10^4^ TCID_50_ of virus could lead to more severe symptoms and earlier death. Although the viral load in the 10^4^ TCID_50_ group peaked faster than that in the 10^2^ TCID_50_ group, the peak viral load seemed not to be related to the viral inoculum.

### ZIKV displayed distinct replication dynamics and tissue tropism in AG129 mice

**Fig. 2:**
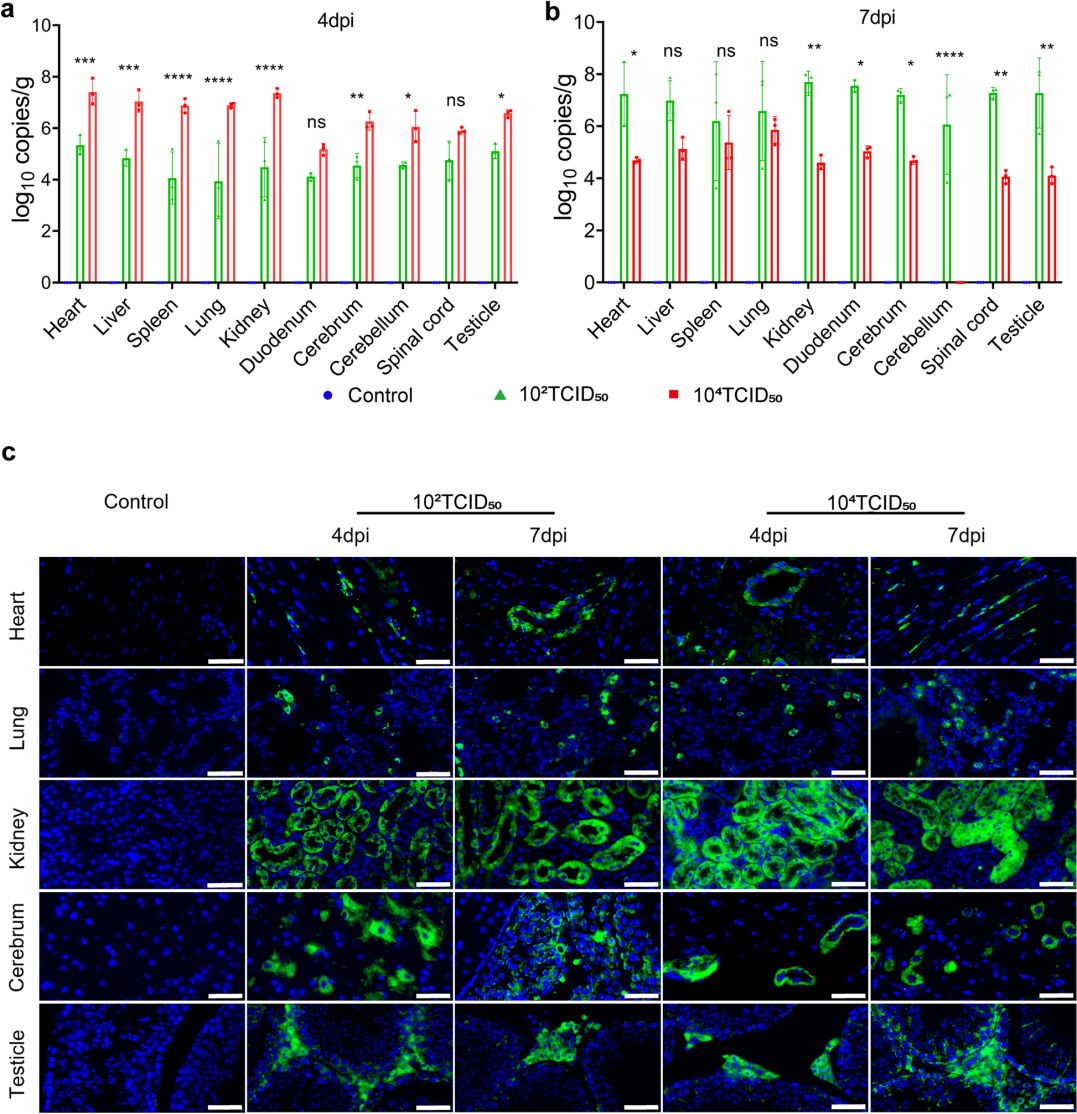
ZIKV displayed distinct replication dynamics and tissue tropism in AG129 mice. **a** Viral load in heart, liver, spleen, lung, kidney, duodenum, cerebrum, cerebellum, spinal cord, and testicle of three groups of AG129 mice at 4 dpi (*n* = 3); **b** Viral load in heart, liver, spleen, lung, kidney, duodenum, cerebrum, cerebellum, spinal cord, and testicle of three groups of AG129 mice at 7 dpi (*n* = 3); **c** Immunofluorescence image of heart, lung, kidney, cerebrum, and testicle of three groups of AG129 mice at 3 dpi and 7 dpi (*n* = 3). Zika virus capsid protein antibody is used as the first antibody. Green represents ZIKV-infected cells and blue represents DAPI staining. The scale bar represents 50 µm. Quantitative data are shown as the mean ± SD (error bars). *P < 0.05, **P < 0.01, ***P < 0.001, ****P < 0.0001 (Sidak's multiple comparisons test)

According to previous research, ZIKV exhibits extensive tissue tropism and the ability to replicate in blood, which could affect the function of many organs [17, 29]. Mice of each group were euthanized humanely for tissue sampling at 4 dpi and 7 dpi to evaluate any potential ZIKV RNA in anatomic tissues, including heart, liver, spleen, lung, kidney, duodenum, cerebrum, cerebellum, spinal cord and testicle. We detected the high levels of viral RNA expression in these tissues by RT-qPCR. What is noticeable is that the viral load of most tissues in the 10^4^ TCID_50_ group is higher than that in the 10^2^ TCID_50_ group at 4dpi (Fig. 2a), while the viral load of most tissues in the 10^4^ TCID_50_ group is lower than that in the 10^2^ TCID_50_ group at 7dpi (Fig. 2b). In addition, the viral load of these tissues at 4 dpi is lower than that at 7 dpi in the 10^2^ TCID_50_ group, while the viral load of these tissues at 4 dpi is higher than that at 7 dpi in the 10^4^ TCID_50_ group. The change of viral load in these tissues is consistent with that in blood (Fig. 1c). Meanwhile, we detected viral titer in some tissues of each group at 7 dpi, including heart, lung, kidney, cerebrum, testicle, and blood (Fig. S2). The results indicated that there were infectious viruses in all these tissues. Therefore, we performed immunofluorescence assays of these tissue slices to observe more about the localization and distribution of viral antigens (Fig. 2c).

### Pathological lesions appeared in tissues with infectious virus

**Fig. 3:**
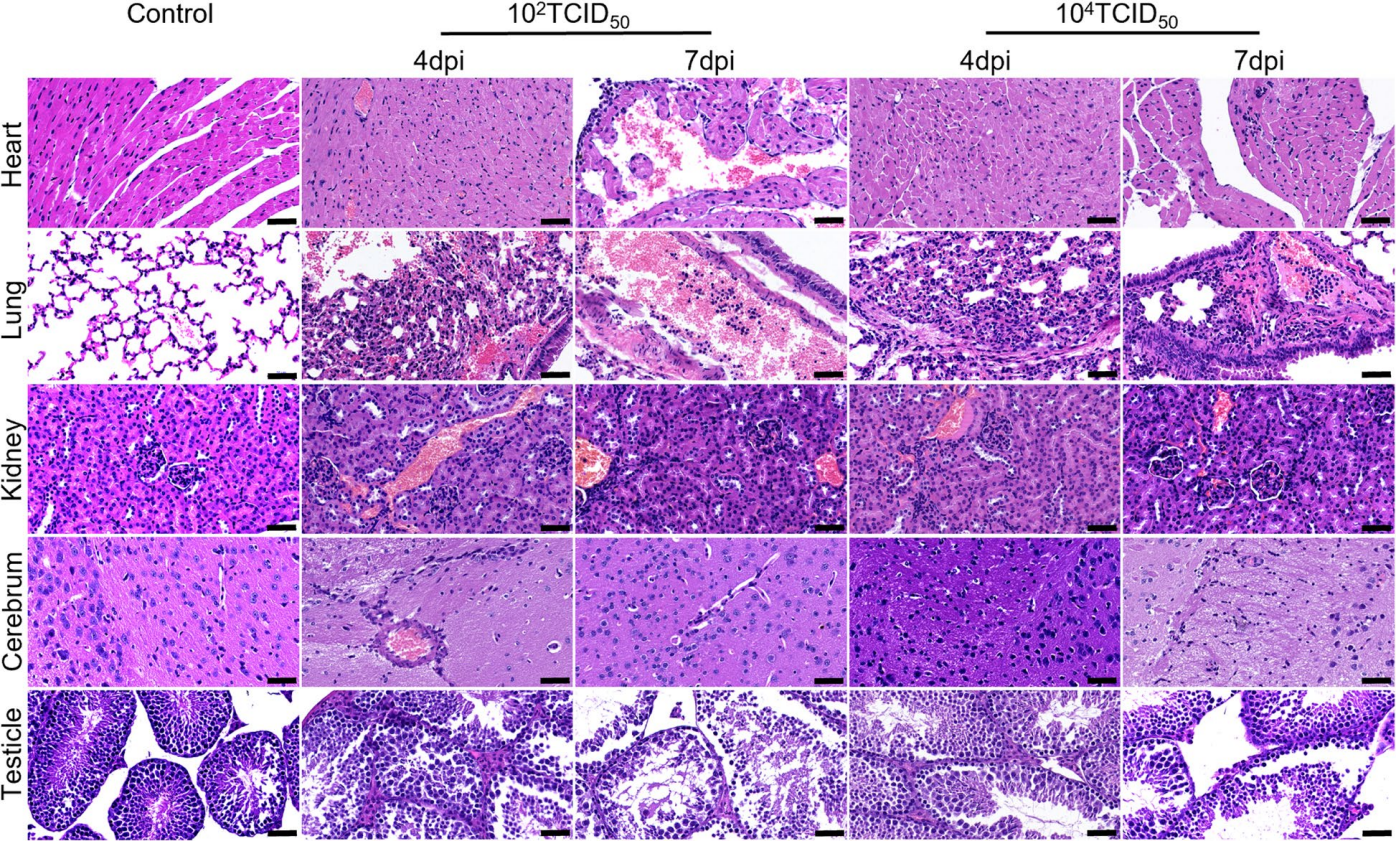
Pathological lesions appeared in tissues with infectious virus. Pathological lesions were observed with H&E stained slides (*n* = 3). The scale bar represents 50 µm

To gain insight into the impact of ZIKV infection on heart, lung, kidney, cerebrum, and testicle, these tissue samples were obtained at 4 dpi and 7 dpi for histopathologic analyses (Fig. 3). Compared to the control group, we observed extensive ZIKV-associated pathological lesions in challenged groups. In the myocardium, the inflammatory cells increased, and the local congestion was mild; In addition, pathological lesions of the 10^2^ TCID_50_ group at 7 dpi were relatively severe. In lung tissue after virus infection, inflammatory cells increased significantly with alveolar septum hemorrhage, pulmonary septum thickening, and bronchial mucosa shedding, and hemorrhage and inflammatory infiltration of the 10^2^ TCID_50_ group at 7 dpi were more severe. In kidney tissue after virus infection, pathological lesions mainly included vascular congestion, mild local bleeding, and inflammatory cell increase. In the cerebrum, microglia increased. In testicle, tissue was loose, and spermatogonium decreased. Meanwhile, we also found pathologic lesions in liver, spleen, and duodenum (Fig. S3), though no infectious virus was detected in these tissues. In liver, lymphocyte infiltration and mild congestion were observed locally. In spleen, the germinal center disappeared with lymphocyte infiltration and local congestion. In duodenum, lymphocytes increased with local mucosal structure damage.

### ZIKV infection resulted in a massive release of cytokines at 4dpi in AG129 mice

**Fig. 4:**
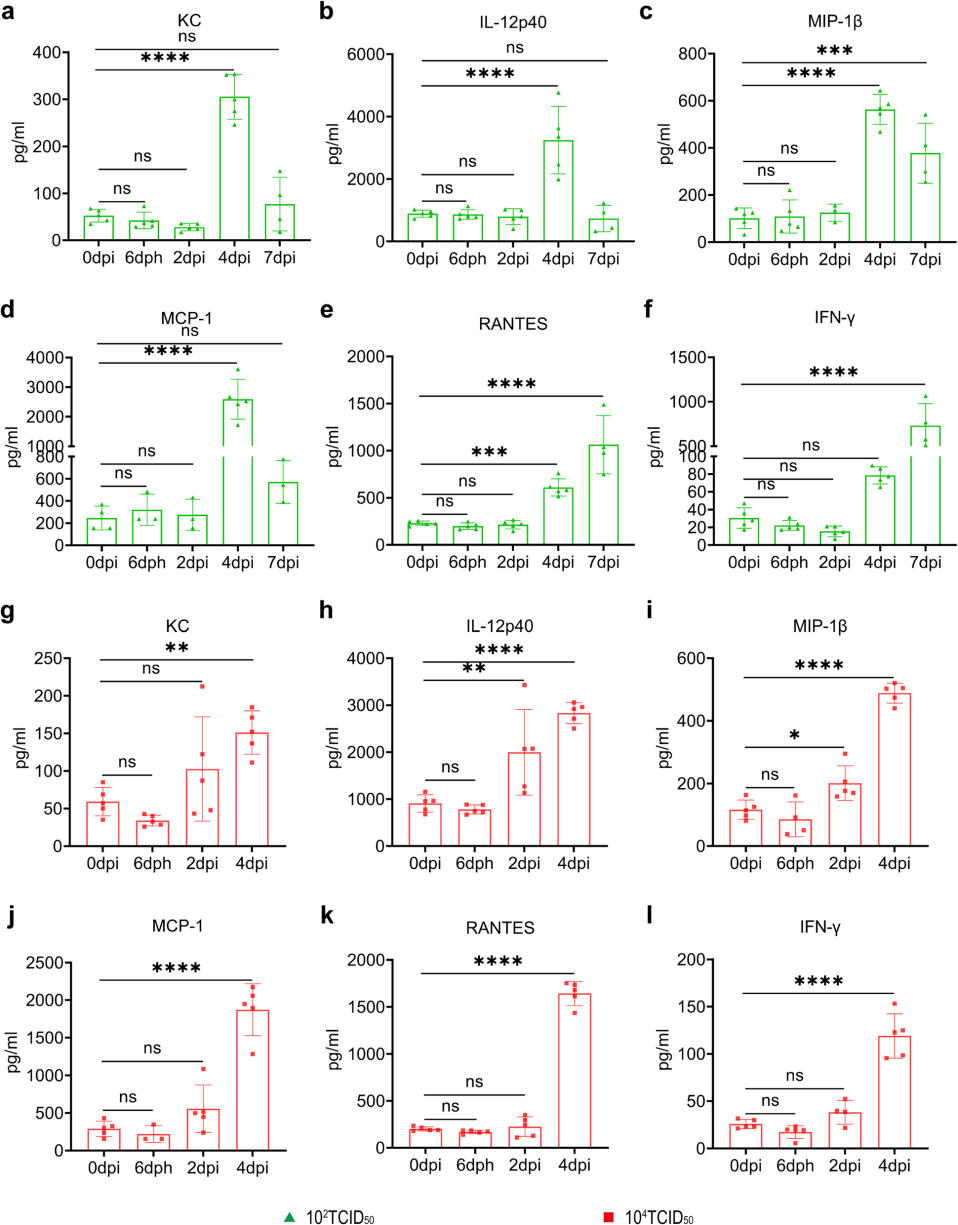
ZIKV infection resulted in a massive release of cytokines at 4dpi in AG129 mice. **a-f** the expression of KC (**a**), IL-12p40 (**b**), MIP-1β (**c**), MCP-1 (**d**), RANTES (**e**) and IFN-γ (**f**) changed in AG129 mice infected with 10^2^ TCID_50_ of ZIKV (*n* = 5); **g-l** the expression of KC (**g**), IL-12p40 (**h**), MIP-1β (**i**), MCP-1 (**j**), RANTES (**k**) and IFN-γ (**l**) changed in AG129 mice infected with 10^4^ TCID_50_ of ZIKV (*n* = 5). Quantitative data are shown as the mean ± SD (error bars). *P < 0.05, **P < 0.01, ***P < 0.001, ****P < 0.0001 (one-way ANOVA)

To characterize immune activation during ZIKV infection, we compared the change of serum levels of several cytokines, chemokines, and growth factors in AG129 mice of the infection groups. The massive release of KC, IL-12p40, MIP-1β, MCP-1 and RANTES happened on the fourth day after ZIKV infection (Fig. 4a-e, Fig. 4g-k). the massive expression of IFN-γ and G-CSF in the serum of 10^4^ TCID_50_ group was detected early at 4 dpi (Fig. 4l, Fig. S4g), while that of 10^2^ TCID_50_ group was detected at 7 dpi (Fig. 4f, Fig. S4b). The expression of KC in the serum of 10^2^ TCID_50_ group is higher than that of 10^4^ TCID_50_ group at 4dpi (Fig. 4a,g). On the contrary, the expression of RANTES, IFN-γ, eotaxin and G-CSF in the serum of 10^2^ TCID_50_ group is lower than that of 10^4^ TCID_50_ group at 4dpi (Fig. 4e,f,k,l, Fig. S4a,b,f,g). There was no significant difference in the expression of IL-12p40, MIP-1β and MCP-1 in serum of these two infection groups at 4 dpi. ZIKV infection similarly did not affect the expression level of IL-12p70 and TNF-α (Fig. S4d,e,i,j).

### Favipiravir showed antiviral effect in ZIKV-challenged AG129 mice

**Fig. 5:**
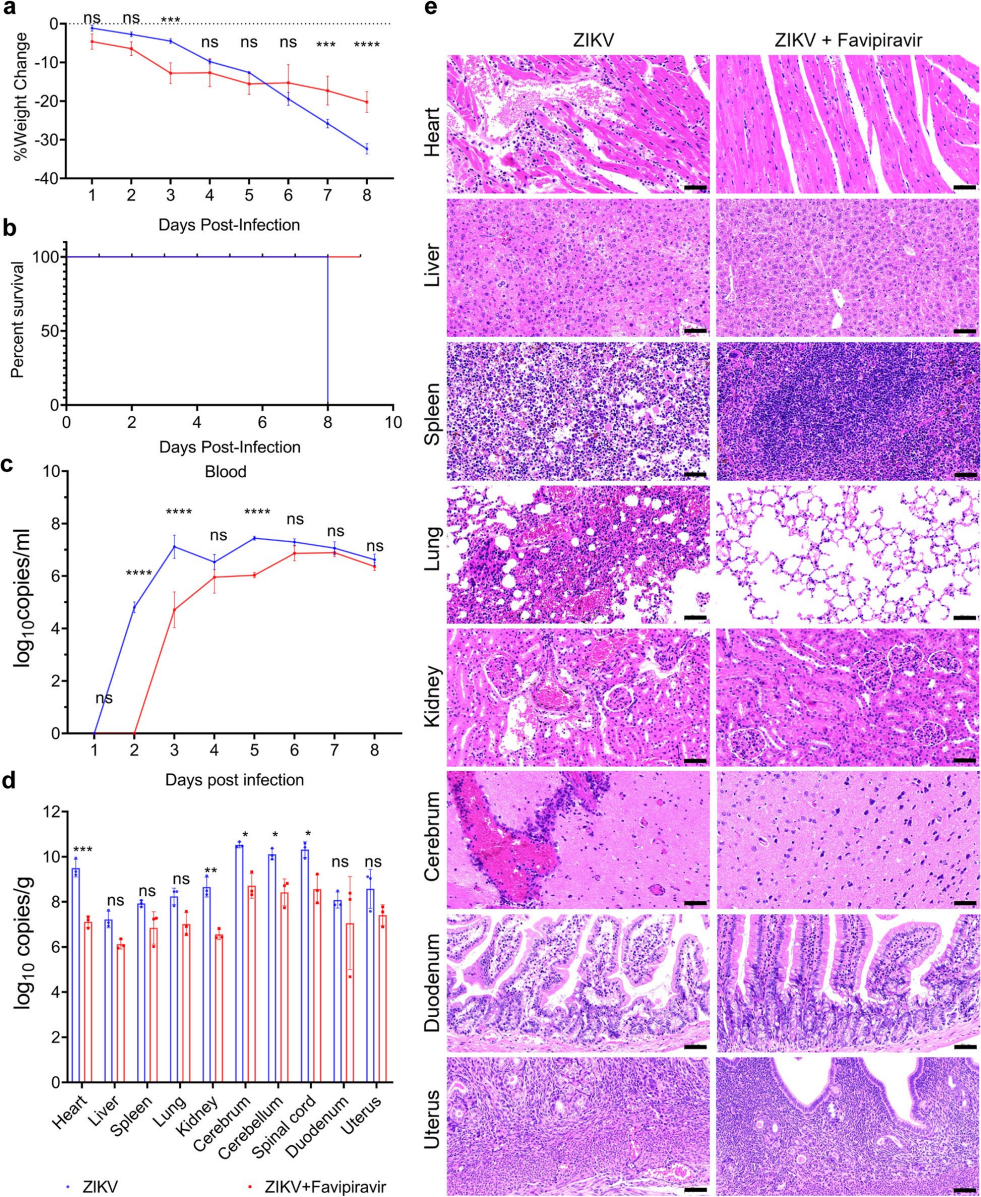
Favipiravir showed antiviral effect in ZIKV-challenged AG129 mice. **a** Line chart of weight change of AG129 female mice of ZIKV group and favipiravir group measured from 0 dpi to 8 dpi (*n* = 3); **b** Survival percentage for AG129 female mice of ZIKV group and favipiravir group (*n* = 3); **c** Line chart of viral load in blood of AG129 female mice of ZIKV group and favipiravir group measured from 0 to 8 dpi (*n* = 3); **d** Viral load in heart, liver, spleen, lung, kidney, duodenum, cerebrum, cerebellum, spinal cord, and uterus of AG129 female mice of ZIKV group and favipiravir group at 8 dpi (*n* = 3). Quantitative data are shown as the mean ± SD (error bars). *P < 0.05, **P < 0.01, ***P < 0.001, ****P < 0.0001 (Sidak's multiple comparisons test); **e** Pathological lesions were observed with H&E staining in heart, liver, spleen, lung, kidney, cerebrum, duodenum and uterus of AG129 female mice of ZIKV group and favipiravir group at 8 dpi (*n* = 3). The scale bar represents 50 µm

To prove the practical value of the AG129 mouse model, we evaluated the antiviral activity of favipiravir against ZIKV (1*10^3^TCID_50_) in female AG129 mice. In the model, we gave mice favipiravir by intragastric administration every day. Favipiravir group displayed a statistically significant reduction of weight compared to the ZIKV group at 7 dpi (Fig. 5a). All untreated mice were immobile and dying at 8 dpi, while all treated mice were alive and active at 9 dpi (Fig. 5b). Viral load in blood of favipiravir group rose more slowly than that of ZIKV group (Fig. 5c). The statistical reduction of viral load in favipiravir group emerged in heart, kidney, cerebrum, cerebellum and spinal cord (Fig. 5d). According to pathological outcome (Fig. 5e), the symptoms of bleeding or inflammatory cell infiltration are milder in the myocardium, liver, spleen, lung, kidney, cerebrum, duodenum and uterus of favipiravir group than ZIKV group. In addition, the pathological changes of myocardial fibrosis, bronchial obstrction, the loss of germinal centers and intestinal mucosal injury were milder in favipiravir group than ZIKV group. We could notice that the difference of pathological lesions between these two groups appeared in heart, liver, spleen, lung, kidney, cerebrum and duodenum based on the pathological score (Fig. S6a). Meanwhile, we also evaluated the antiviral activity of testosterone against ZIKV (1*10^3^TCID_50_) in male AG129 mice. In the model, we gave mice testosterone by intraperitoneal injection at 2 dpi and 4 dpi. Testosterone group did not show significant antiviral efficacy compared to the ZIKV group according to the results of weight change, survival rate and viral load (Fig. S5a-d). According to the pathological changes (Fig. S5e) and pathological score (Fig. S6b), the symptoms of bleeding and inflammatory cell infiltration are milder in the testicle of testosterone group than ZIKV group. However, we could not observe significant differences in other tissues of these two groups.

## Discussion

Mice are the most widely used laboratory animals due to their low cost of breeding, short generation time, and small size. Interestingly, the natural host of the Zika virus comprises mainly mosquitoes, nonhuman primates, and humans [30]. The virus could not infect wild-type mice or immunocompetent mice due to the species-restricted difference in antiviral IFN signaling pathway [26, 31]. The double knockout of IFN-α/β and IFN-γ receptors abolishes innate immunity that prevents ZIKV infection early. Thus, ZIKV could efficiently infect immunocompromised mice that are deficient in IFN-α/β and IFN-γ receptors (AG129 mice) [32]. In this study, we chose AG129 mice as a small animal model to explore the infection characteristics, viral dynamics, pathological features, and cytokines change after ZIKV challenge.

During ZIKV infection, some cells and tissues may become viral reservoirs, contributing to the dissemination of ZIKV to nearby tissues. We have detected viral load in many tissues, including heart, liver, spleen, lung, kidney, duodenum, cerebrum, cerebellum, spinal cord, and testicle, which indicated extensive tissue tropism and was consistent with previous research [33, 34]. It is noteworthy that the viral load in these tissues of the 10^4^ TCID_50_ group at 4dpi is higher than that of the 10^2^ TCID_50_ group, but the viral load in these tissues of the 10^4^ TCID_50_ group at 7dpi is lower than that of the 10^2^ TCID_50_ group. Interestingly, viral load in these tissues of the 10^2^ TCID_50_ group increased by approximately 1–4 logs from 4 to 7 dpi, while the viral load in these tissues of the 10^4^ TCID_50_ group decreased by approximately 0–6 logs during the same time. These results demonstrated viral RNA levels are specific to the injection dose, time point, and tissue type. Meanwhile, we have also detected infectious viruses in some tissues at 7 dpi including heart, lung, kidney, cerebrum, testicle, and blood. As is known to all, the brain and testicle belong to immune-privileged organs. The immune-privileged nature may allow ZIKV to persist in these tissues once the virus enters [35, 36]. In our study, we not only detected viral load by RT-qPCR in the cerebrum and testicle but also infectious virus, which is consistent with previous studies in rhesus macaques and mice [32, 35–38]. While all of these tissues were found to be susceptible to ZIKV infection, infectious viruses were noticed to be the lowest in the cerebrum. ZIKV replication is cytopathic in neuronal cells, which could cause neurological damage in newborns [39]. These results proved that ZIKV could cross the blood–brain barrier and blood-testis barrier and further confirmed that the virus had the ability of neurotropism and sexual transmission [40–43]. Previous studies suggest that multiple candidate receptors for ZIKV are not specifically expressed in these tissues, which may explain ZIKV’s broad tissue tropism [44].

In addition, these tissues in which infectious virus were detected have appeared histopathological lesions attributable to infection with ZIKV. ZIKV could uniquely manipulate the host machinery to promote viral replication in a tissue-specific manner, resulting in characteristic pathological disorders [45]. It is noteworthy that related lesions appeared in the cerebrum and testicle. We did not observe more obvious pathological changes associated with microcephaly and male infertility. It is probably because microcephaly happened in fetal mice infected with ZIKV during pregnancy [46]. In a related mouse model, ZIKV infection causes complete destruction of the testicular structure up to two months after infection and leads to male infertility [35]. While our AG129 mice infected with 10^2^ TCID_50_ of GZ01 died at 8 ~ 9 dpi. Although there was no infectious virus detected in the liver, spleen, and duodenum, pathological lesions happened in these tissues, which might be either due to the focal nature of infection, detection sensitivity or viral clearance during the necropsy of mice or because of a massive release of cytokines activated by ZIKV infection. Therefore, this dysregulated immune response led to multiple organ damage or death, especially liver with ample blood supply. Previous studies have demonstrated that the spleen and intestine as the lymphoid-rich organs were potential targets of ZIKV dissemination [47–49]. Our results supported that the levels of cytokines (KC, IL-12p40, MIP-1β, MCP-1, RANTES) in blood increased significantly at 4 dpi, indicating a potential role for these immune-related factors in ZIKV pathogenesis [20, 50]. Moreover, the expression level of several cytokines (KC, IL-12p40, MIP-1β, MCP-1) showed a similar trend with viral load in blood of AG129 mice. These cytokines might join in antiviral response at 3 dpi or 4 dpi. Viral load in blood of 10^4^ TCID_50_ group decreased faster than that of 10^2^ TCID_50_ group, which might be attributable to the higher expression of RANTES, IFN-γ, eotaxin and G-CSF in 10^4^ TCID_50_ group than 10^2^ TCID_50_ group at 4 dpi. It is noteworthy that the expression of KC of 10^2^ TCID_50_ group is higher than that of 10^4^ TCID_50_ group. Combined with more severe leision in heart and lung of 10^2^ TCID_50_ group at 7 dpi, it might be KC that incurred more severe inflammatory response. Importantly, inflammatory damage appeared in most tissues of AG129 mice at 4 dpi, which indicated a massive release of cytokines due to excessive activation of immune cells destroyed a fragile equilibrium of pro- and anti-inflammation effect. The degree to which IFN-deficient mice model the extent and severity of ZIKV infection in humans is unknown. We recognize that healthy humans may be able to mount an effective antiviral response and prevent viral entry into the central nervous system. However, it remains a possibility that some immunocompromised humans and even some healthy humans may be susceptible in ways modeled by the AG129 mice.

To support the future value of AG129 mouse model, we chose favipiravir as a drug candidate against ZIKV. To ensure that untreated mice die at 8 dpi, we chose the inoculum dose of 10^3^ TCID_50_. Favipiravir is an RNA polymerase inhibitor. According to experimental results, favipiravir seemed to inhibit the early replication of ZIKV in vivo and could alleviate infection symptoms significantly. Some results support antiviral efficacy of favipiravir against ZIKV in nonhuman primates [51]. Previous research has also proven that testosterone can reduce mortality and attenuate testicular damage in ZIKV-infected A129 mice [52]. However, our models only indicate that the testosterone could relieve ZIKV-induced testicular lesion. We think that the antiviral effect of testosterone may be specific to the ZIKV strains and mouse strains. In addition, the time, frequency, and dose of administration may also affect the experimental results. Therefore, in the future, researchers should focus on the study of pharmacokinetic and pharmacodynamic properties of testosterone in ZIKV-infected AG129 mice and find the optimal dosage regimen.

Therefore, robust animal models are crucial for advancing viral research and screening related drugs. First, our mouse model appeared to reproduce key features of human infection by ZIKV, including viremia, testicular tropism, and neuronal tissue tropism, which is valuable to study fundamental questions about ZIKV pathogenesis. Second, clinical data shows that symptoms associated with ZIKV infection in humans do not appear until 3–11 days after infection [53], which makes it difficult to study early viral infection. However, viral load in the blood of our mouse model could be detected as early as the second day after infection. Third, ZIKV is primarily transmitted to humans by the *Aedes aegypti* mosquito, and our AG129 mice were infected with the virus by subcutaneous injection, which could mimic mosquito biting to study mosquito-to-vertebrate transmission [54]. Fourth, ZIKV challenge with 10^2^ TCID_50_ caused AG129 mice to die early at 8dpi, which demonstrated the virulence of GZ01. Rapidly worsening symptoms could shorten the cycle of drug screening in AG129 mice. Fifth, we observed a massive release of cytokines at 4 dpi, which might be a critical factor resulting in extensive pathological lesions of AG129 mice. The discovery can contribute to clinical decision on how to control the balance of pro- and anti-inflammatory responses during ZIKV infection. Finally, we also have noticed that people previously infected with dengue virus may exacerbate the symptoms of ZIKV infection because of antibody-dependent enhancement of the two viruses [55, 56]. Importantly, AG129 mice are susceptible to both dengue and Zika virus infection [54, 57]. In short, the results presented here establish the outstanding potential of the ZIKV mouse model with unique features—specifically effective infectious dose, tissue tropism, pathological lesion, immune response, and early infection, which could significantly contribute to potential vaccination and antiviral strategies. According to our mouse model, we have discovered that the inoculum dose of Zika virus can affect the viral replication dynamics, cytokine responses and survival rate in AG129 mice and that AG129 mice can be used to screen and evaluate antiviral drugs successfully. However, we did not evaluate the vaccine in the AG129 mouse model, which is what we need to improve in the future. In today's world of close interaction, the virus can spread rapidly from one country to another by international routes, potentially bringing the risk of disease and death to every corner of every continent on Earth. Although there is no sign of another ZIKV pandemic outbreak, we believe it is still necessary to study and improve Zika-related animal models constantly. After all, the Zika virus has the mutational potential for increased pathogenicity and enhanced transmissibility [14], and there are no specific drugs and vaccines to inhibit the Zika virus [58].

## Materials and methods

### Animals, ethics, and biosafety statement

AG129 mice (aged 6 ~ 8 weeks) were purchased from the National Institutes for Food and Drug Control (NIFDC) (manufacturing license: SCXK (JING)2022–0002). All animal procedures were approved by the Institutional Animal Care and Use Committee of the Institute of Medical Biology, Chinese Academy of Medical Science (ethics number: DWSP202108008), and performed in strict accordance with the guidelines for the ABSL-2 facility of the Institute of Medical Biology, Chinese Academy of Medical Science, Yunnan, China.

### Cell and virus

Vero cells were self-stored in our laboratory and cultured in Dulbecco’s modified Eagle medium (DMEM) (Gibco) supplemented with 10% fetal bovine serum (FBS) (Gibco) and 1% penicillin/streptomycin at 37 °C with 5% CO_2_. The ZIKV strain GZ01 (GenBank: KU820898) was obtained from the Institute of Medical Biology, Chinese Academy of Medical Science, and propagated in Vero cells in DMEM (Gibco) supplemented with 2% FBS and 1% penicillin/streptomycin.

#### AG129 mouse experiment

AG129 mice were maintained and bred in a conventional animal facility. All mice were challenged with ZIKV (1*10^2^ and 1*10^4^ TCID_50_) via subcutaneous injection in the inner thigh. We selected eleven animals per inoculum dose. Mice were weighed and monitored daily for signs of disease. For all procedures (such as subcutaneous injection, physical examinations, and blood sampling), the animals were anesthetized via inhalation of vaporized isoflurane. Three AG129 mice in each group were sacrificed at 4 dpi and 7 dpi for tissue sample collection. Blood samples were collected from five AG129 mice in each group at 0 dpi, 6 h post infection (hpi), 2 dpi, 4 dpi and 7 dpi.

#### Viral load

Viral RNA was extracted with a KingFisher Flex Purification System (Thermo Fisher) and a MagMAX™-96 Viral RNA Isolation Kit (Thermo Fisher AM1836). Viral RNA isolated from different tissues was quantified by RT‒qPCR, which was performed using TaqMan Fast Viral 1-Step Master Mix (Thermo Fisher, 4,444,432) on a CFX384 Touch Real-Time PCR Detection System (Bio-Rad, USA). Forward primer (5′-TTGGTCATGATACTGCTGATTGC-3′), reverse primer (5′-CCTTCCACAAAGTCCCTATTGC-3′) and probe (5′-FAM-CGGCATACAGCATCAGGTGCATAGGAG-BHQ1-3′) targeting the ZIKV prM-E gene were used. The reaction system consisted of 2.5 μL qPCR mix, 0.5 μL forward primer, 0.5 μL reverse primer, 0.5 μL probe, 3.5 μL water, 2.5 μL RNA sample, which were added to each well of a 384-well PCR plate. The cycling conditions were as follows: 50 °C for 5 min, 95 °C for 20 s, and 40 cycles of 95 °C for 3 s and 60 °C for 30 s. After the reaction finished, the data were collected, and the number of viral copies was calculated based on the standards.

#### Viral titration

The virus concentration was quantified as the median tissue culture infective dose (TCID_50_) in Vero cells. The cells were inoculated with six tenfold serial dilutions of isolated serum from daily blood in DMEM supplemented with 2% fetal bovine serum and 1% penicillin/streptomycin. The cells were incubated for six days. The TCID_50_ was calculated via the Karber formula.

#### Immunofluorescence assay

Paraffin sections were sequentially soaked in xylene I, xylene II, xylene III, 100% ethanol, 100% ethanol, 95% ethanol, 85% ethanol, and 75% ethanol, followed by washing three times in PBS. Next, the sections were boiled in sodium citrate buffer in a microwave for 15 min. After the samples were soaked and washed twice in PBS, they were transferred to 0.5% TritonX-100 for permeation and incubated at room temperature for 10 min. Then, the slices were incubated with blocking buffer at room temperature for one hour. After that, Zika virus capsid protein antibody (GeneTex, Irvine, CA, USA, GTX133317) was added to the slices, which were incubated overnight at 4 ℃, followed by soaking and washing three times. Then, goat anti-rabbit IgG H&L conjugated with Alexa Fluor® 488 (Abcam, ab150081) was added as a secondary antibody, and the samples were incubated in the dark for one hour at room temperature, followed by soaking and washing three times. The coverslips were covered after the addition of antifade mounting medium containing DAPI, and the slides were observed under a fluorescence microscope (Leica).

#### Histopathology

For histopathological evaluation, tissue samples were fixed in 10% formalin and embedded in paraffin. Serial tissues were cut into 5-μm sections and stained with hematoxylin and eosin (HE). These slides were scanned with 3DHISTECH. H&E stained slides were scored by an experienced pathologist using CaseViewer from the manufacturer.

#### Cytokine measurement

To determine the levels of immune-related factors, we used a Bio-Plex Pro Mouse Cytokine 23-plex Assay #M60009RDPD and performed detection according to the manufacturer’s instructions. Before running the assay, the total number of wells in the experiment was determined, and we calculated the required volumes of coupled beads and detection antibodies. Then, the blood was allowed to clot for at least one hour at room temperature before centrifugation for 15 min at 3500 rpm, and the serum was transferred to a clean polypropylene tube. Next, the coupled beads, samples, standards, blanks, detection antibodies, and streptavidin–phycoerythrin were added to each well according to the manufacturer’s instructions. Finally, the plate was read immediately, and the data were processed.

#### Drug evaluation

We chose male AG129 mice for testing testosterone and female AG129 mice for testing favipiravir. Three mice in each group were prepared for ZIKV challenge (1*10^3^ TCID_50_). The testosterone group was treated with 0.05 mg of testosterone in 100 ml of sesame seed oil by intraperitoneal injection on days 2 and 4 after ZIKV infection [52]. The favipiravir group was treated with 300 mg/kg favipiravir by intragastric administration every day [51]. We recorded the body temperature and weight of these mice daily. Moreover, we collected blood samples to detect viremia until the mice died. Finally, we sacrificed these mice at 9 dpi and collected tissue samples to detect the viral load.

#### Statistical analysis

Statistical analysis was performed with GraphPad Prism (8.0.2). We have described detailed statistics in the figure legends. The following statistical tests were used in this study: Sidak's multiple comparisons test, one-way analysis of variance (ANOVA) and paired t-test. All quantitative data in this study were presented as the mean ± SD. *P* values < 0.05 were considered significant. Significance values were set as follows: NS, not significant, *P* > 0.05; *P < 0.05; **P < 0.01; ***P < 0.001; and ****P < 0.0001.

## Supplementary Information

Supplementary Material 1.

Supplementary Material 2.

## Data Availability

All the data supporting the findings of this current study are available from the corresponding author upon reasonable request.
