# Supplementary material for: The inoculum dose of Zika virus can affect the viral replication dynamics, cytokine responses and survival rate in immunocompromised AG129 mice
Source: Mol Biomed. 2024 Aug 3;5:30. doi: 10.1186/s43556-024-00195-x (PMC11297010; doi:10.1186/s43556-024-00195-x)
Supplement: Supplementary file 1 — Supplementary Material 1. [file 43556_2024_195_MOESM1_ESM.docx]

Supplementary Materials for

The inoculum dose of Zika virus can affect the viral replication dynamics, cytokine responses and survival rate in immunocompromised AG129 mice

**Author information**

Yuhuan Yan ^1#^, Hao Yang^1#^, Yun Yang^1^, Junbin Wang^1^, Yanan Zhou^1^, Cong Tang^1^, Bai Li^1^, Qing Huang^1^, Ran An^1^, Xiaoming Liang^1^, Dongdong Lin^1^, Wenhai Yu^1*^, Changfa Fan^2*^, Shuaiyao Lu^1,3,4,5*^

Author affiliations: 1. Institute of Medical Biology, Chinese Academy of Medical Sciences and Peking Union Medical School, Kunming 650118, China; 2. National Rodent Laboratory Animal Resources Center, Institute for Laboratory Animal Resources, National Institutes for Food and Drug Control (NIFDC), Beijing 102629, China; 3. Key Laboratory of Pathogen Infection Prevention and Control (Peking Union Medical College), Ministry of Education, Beijing, China; 4. State Key Laboratory of Respiratory Health and Multimorbidity, Beijing, China; 5. Yunnan Provincial Key Laboratory of Vector-borne Diseases Control and Research,, Kunming, China

# These authors contribute equally.

* The correspondence author: Shuaiyao Lu, [lushuaiyao-km@163.com](mailto:lushuaiyao-km@163.com); Changfa Fan, [fancf@nifdc.org.cn](mailto:fancf@nifdc.org.cn); Wenhai Yu, Wenhaiyu1234@163.com

**This word file includes:**

Figures. S1, S2, S3, S4 and S5.

Figure S1


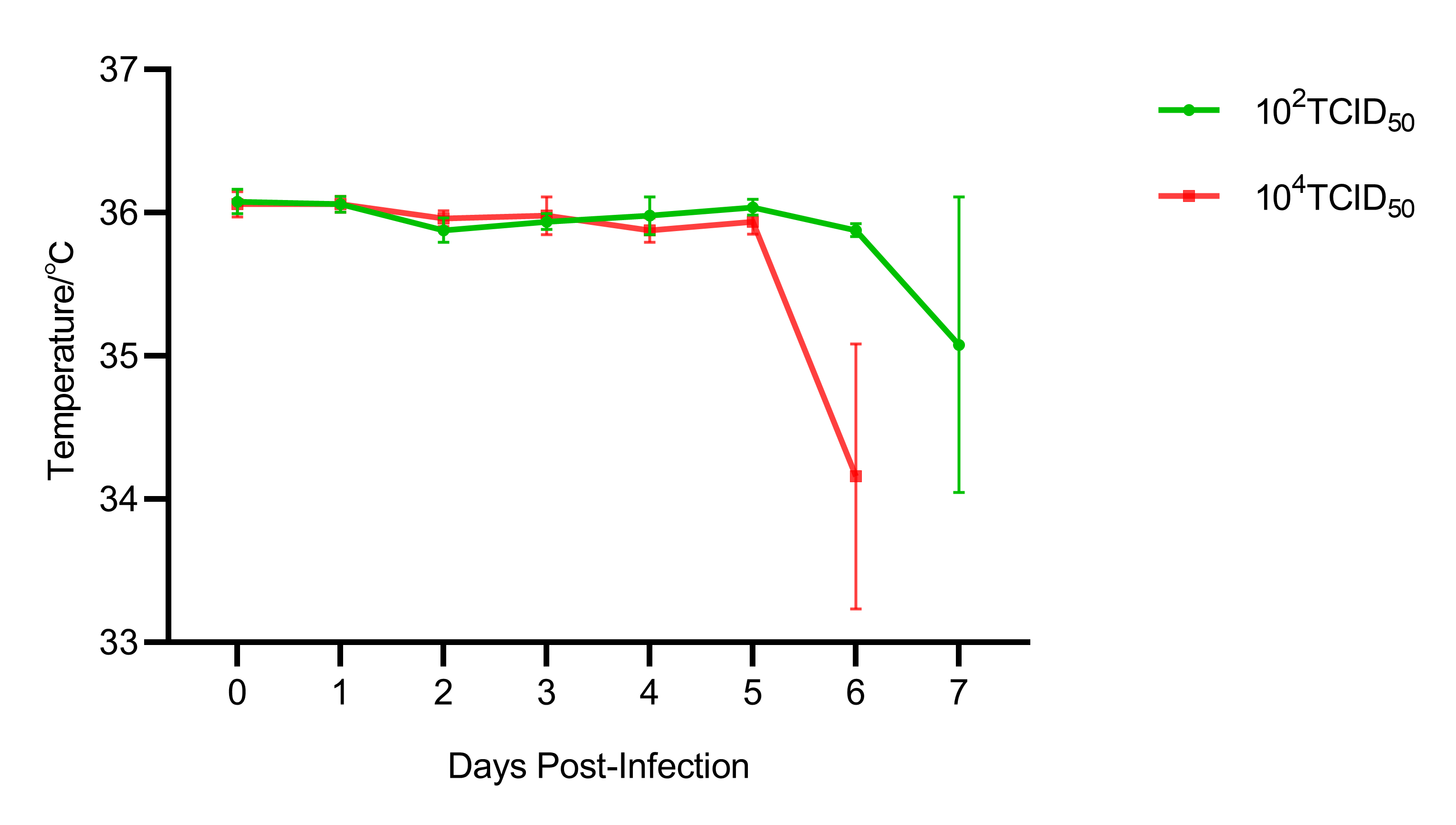


**Fig. S1** Body temperature of AG129 male mice infected with ZIKV did not show regular change from o dpi to 7 dpi (n=5)

Figure S2


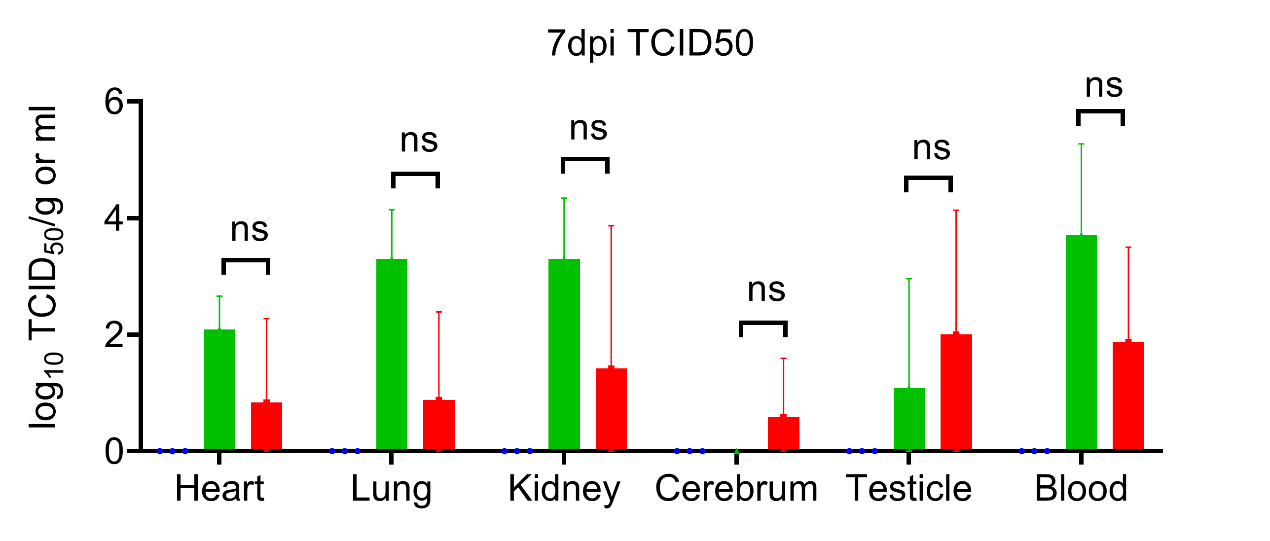


**Fig. S2** Infectiou virus were detected in the heart, lung, kidney, cerebrum, testicle and blood of AG129 male mice at 7 dpi (n=3)

Figure S3


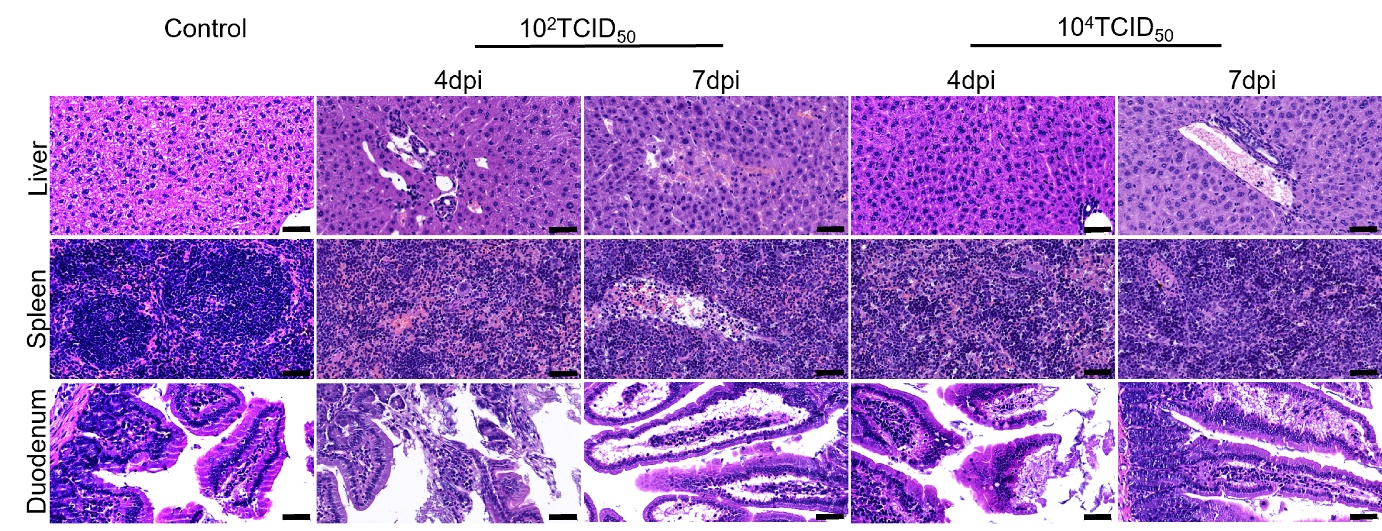


**Fig.S3** Pathological lesions appeared in the liver, spleen and duodenum (n=3, the scale bar represents 50 µm)

Figure S4


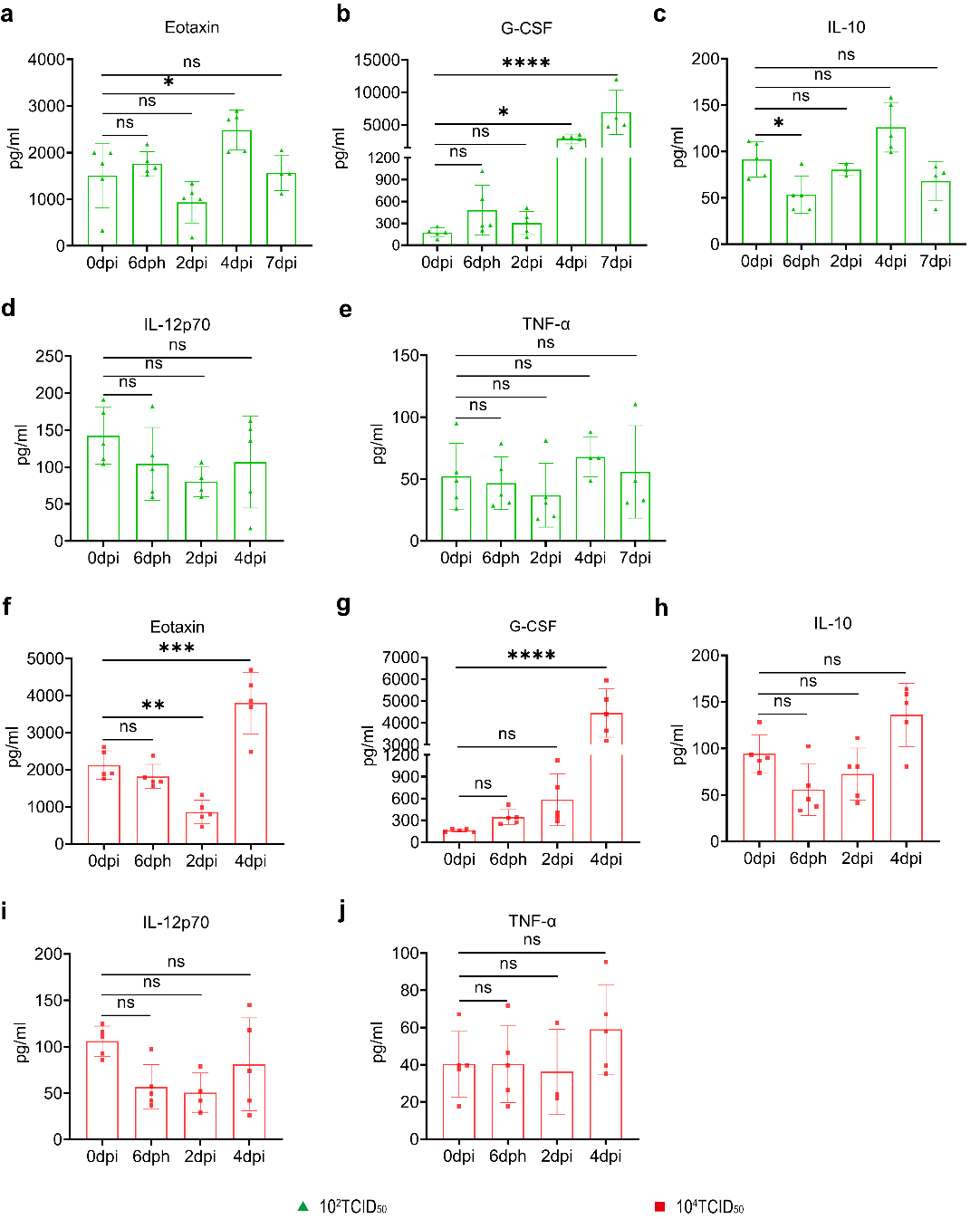


**Fig. S4** The expression of several cytokines during ZIKV infection in AG129 mice

**a-e** the expression of eotaxin (a), G-CSF (b), IL-10 (c), IL-12p70 (d) and TNF-α (e) in AG129 mice infected with 10^2^ TCID_50_ of ZIKV (n=5);

**f-j** the expression of eotaxin (f), G-CSF (g), IL-10 (h), IL-12p70 (i) and TNF-α (j) in AG129 mice infected with 10^4^ TCID_50_ of ZIKV (n=5). Quantitative data are shown as the mean ± SD (error bars). *P < 0.05, **P < 0.01, ***P < 0.001, ****P < 0.0001 (one-way ANOVA)

Figure S5


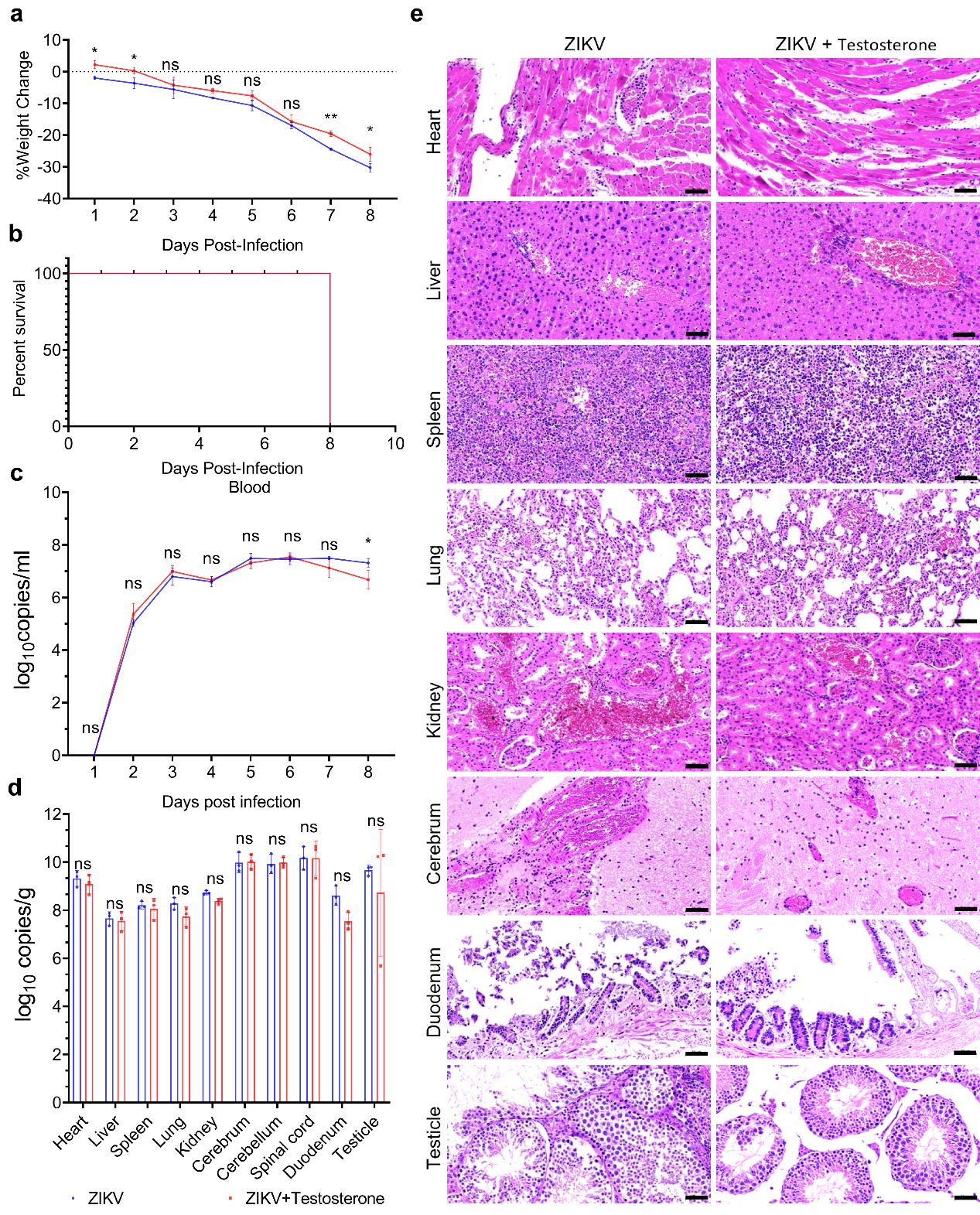


**Fig. S5** Testosterone did not show antiviral effect in GZ01-challenge AG129 mice

**a** Line chart of weight change of AG129 male mice of ZIKV group and testosterone group measured from o dpi to 8 dpi (n=3);

**b** Survival percentage for AG129 male mice of ZIKV group and testosterone group (n=3);

**c** Line chart of viral load in blood of AG129 male mice of ZIKV group and testosterone group measured from 0 dpi to 8 dpi (n=3);

**d** Viral load in heart, liver, spleen, lung, kidney, duodenum, cerebrum, cerebellum, spinal cord, and testicle of AG129 male mice of ZIKV group and testosterone group at 8 dpi (n=3). Quantitative data are shown as the mean ± SD (error bars). *P < 0.05, **P < 0.01, ***P < 0.001, ****P < 0.0001 (Sidak's multiple comparisons test)

e Pathological lesions were observed with H&E staining in heart, liver, spleen, lung, kidney, cerebrum, duodenum and testicle of AG129 male mice of ZIKV group and testosterone group at 8 dpi (n=3). The scale bar represents 50 µm

Figure S6


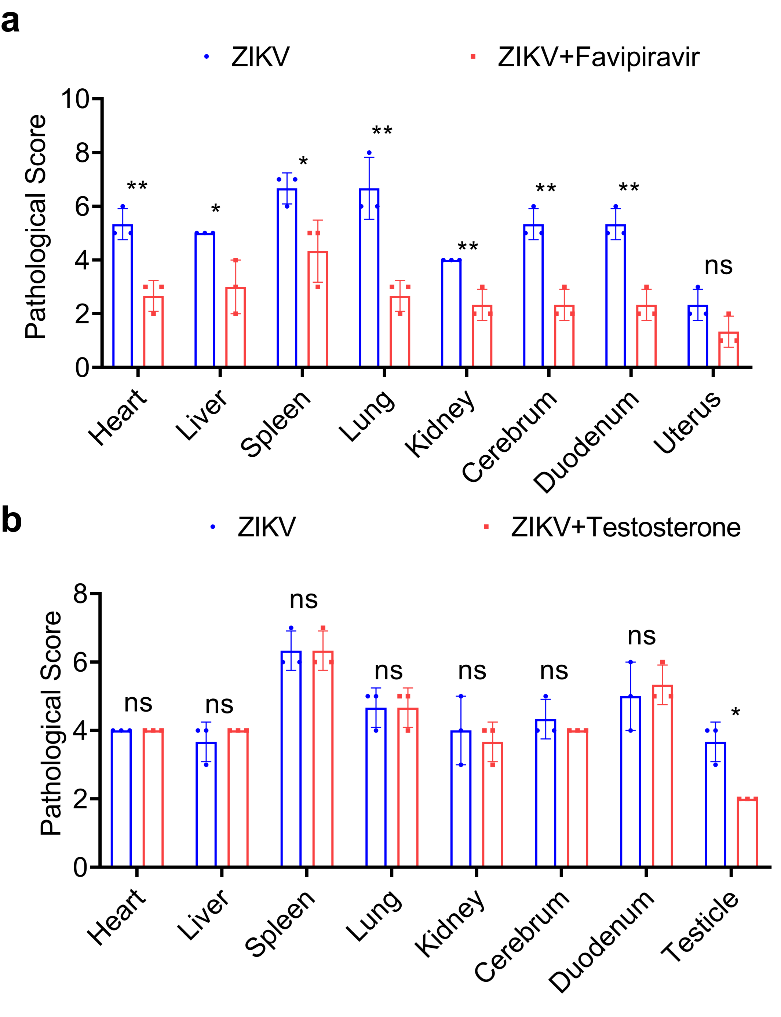


Fig. S6 The pathological score of favipiravir group and testosterone group compared to ZIKV group (n=3). Quantitative data are shown as the mean ± SD (error bars). *P < 0.05, **P < 0.01, ***P < 0.001, ****P < 0.0001 (paired t-test)
