# Supplementary material for: The inoculum dose of Zika virus can affect the viral replication dynamics, cytokine responses and survival rate in immunocompromised AG129 mice
Source: Mol Biomed. 2024 Aug 3;5:30. doi: 10.1186/s43556-024-00195-x (PMC11297010; doi:10.1186/s43556-024-00195-x)
Supplement: Supplementary file 2 — Supplementary Material 2. [file 43556_2024_195_MOESM2_ESM.docx]

IMPORTANT NOTICE: This license only applies if you downloaded this content as

an unsubscribed user. If you are a premium user (ie, you pay a subscription)

you are bound to the license terms described in the accompanying file

"License premium.txt".

---------------------

You must attribute the image to its author:

In order to use a content or a part of it, you must attribute it to Freepik,

so we will be able to continue creating new graphic resources every day.

How to attribute it?

For websites:

Please, copy this code on your website to accredit the author:

<a href="http://www.freepik.com">Designed by Freepik</a>

For printing:

Paste this text on the final work so the authorship is known.

- For example, in the acknowledgements chapter of a book:

"Designed by Freepik"

You are free to use this image:

- For both personal and commercial projects and to modify it.

- In a website or presentation template or application or as part of your design.

You are not allowed to:

- Sub-license, resell or rent it.

- Include it in any online or offline archive or database.

The full terms of the license are described in section 7 of the Freepik

terms of use, available online in the following link:

http://www.freepik.com/terms_of_use

The terms described in the above link have precedence over the terms described

in the present document. In case of disagreement, the Freepik Terms of Use

will prevail.
